# The influence of foreign textile bodies from military clothes on the healing process of experimental injuries of soft tissues

**DOI:** 10.1097/j.pbj.0000000000000145

**Published:** 2022-05-18

**Authors:** Rostislav Mikhaylusov, Vladimir Negoduyko, Sergey Pavlov, Olga Litvinova, Nataliia Babenko, Marina Kumetchko

**Affiliations:** a Kharkiv Medical Academy of Postgraduate Education; b Military Medical Clinical Center of the Northern Region of the Ministry of Defense of Ukraine, Ukraine.

**Keywords:** foreign bodies, inflammation, regeneration, wound healing

## Abstract

**Background:**

The healing of combat wounds can be complicated by the presence of foreign bodies, including fragments of military clothing. The present work aims to study the morphological features of soft tissue injuries with textile fibers implanted into wounds, personnel military forms during wound healing, in the experiment.

**Methods:**

By randomization, 54 rats were divided into 3 groups. Control group animals performed a layer-by-layer incision of soft tissues without implantation of foreign bodies. Animals of the experimental group 1 were made implantation of fibers of a fabric consisting of 100% cotton, and of the experimental group 2–of fibers of a fabric consisting of 65% cotton and 35% polyester. Removal of laboratory animals from the experiment was carried out on the 15th, 30th, and 60th day. Soft tissue samples were histologically examined.

**Results:**

The least pronounced inflammation was observed in rats of the control group. Wound healing in the experimental groups was slowed down due to the presence of inflammatory foci. A more pronounced inflammatory reaction was characterized by a group of animals with implanted tissue fibers consisting of 100% cotton. In the group with implanted tissue fibers consisting of 65% cotton and 35% polyester, the inflammatory reactions were less pronounced.

**Conclusions:**

The presence of textile foreign bodies hampers the healing process of wounds of soft tissues due to the developing processes of inflammation around foreign bodies. The uniform of servicemen (35% synthetic and 65% natural fiber) is less reactive, leaving a wound as a textile foreign body, and has a less pronounced inflammatory effect, apparently due to the presence of synthetic threads that are more inert compared to fabric containing 100% natural fiber. This confirms the need for thorough debridement of combat wounds during the primary surgical treatment.

## Introduction

Over the last few decades, there has been an increase in soft tissue damage using military weapons.^1^ This is due to the increased frequency of religious, ethnic, social conflicts, and the extensive use of weapons during military, peacekeeping missions, and operations.^2^

Wounds received from modern firearms have a pronounced damaging and destructive effect.^3^ Therefore, to develop the most effective treatment plan for combat wounds, it is necessary to use a holistic approach aimed at creating conditions for a favorable course of the wound process.^4^ Comprehensive treatment of combat wounds implies continuous improvement and development of new methods for the diagnosis and treatment of injuries.^5^,^6^

Presence of foreign bodies’ in the wound channel, which falls under the direct penetrating action of the injuring projectile and the vortex flow formed when the injuring projectile passes through the tissue is one of the factors influencing the further healing of combat wounds.^7^,^8^ Most often, in the wound channel there are metallic ferromagnetic foreign bodies in the form of bullets or fragments.^8^ However, there are also foreign bodies in the form of wood chips, glass fragments, soil, and textile tissue fragments. Among textile foreign bodies, fragments of personnel military uniforms are most common.^9^ Often, the foreign object cannot be easily found or deleted. According to the literature, the long-term presence of foreign bodies in soft tissues leads to the formation of a capsule with the presence of a chronic inflammatory process with impaired repair and regeneration.^10^,^11^,^12^

However, there is no unambiguous information in the modern literature about the morphological changes of features of soft tissues in the presence of foreign bodies in case of injuries. Thus, the relevance of this study is due to pathogenic and clinical problems, the solution of which will significantly improve the results of diagnosis and treatment of combat wounds with the presence of foreign bodies in the wound canal.

The purpose of the present work was to study the morphological features of soft tissue injuries with textile fibers implanted into wounds, personnel military forms during wound healing, in the experiment.

## Materials and methods

Permission of the Ethics and Bioethics Commission of the Kharkiv Medical Academy of Postgraduate Education dated 11/ 12/2019 was obtained for the study (a research report compiled by the ARRIVE guidelines 2.0).^13^

### Experimental animals

This study was performed on 54 male Wistar rats aged 5 months and weighing 240 ± 30 g. Laboratory animals were kept in the vivarium of the Kharkiv Medical Academy of Postgraduate Education. The conditions of detention complied with international regulations^14^ and included a natural light regime, optimal temperature (20°C–22°C), a standard diet, and free access to water and food. The animal cages were of sufficient size with natural bedding and ventilation. Experimental work was carried out by international requirements for the treatment of the animal.^15^,^16^

### Implantable samples

**Figure 1:**
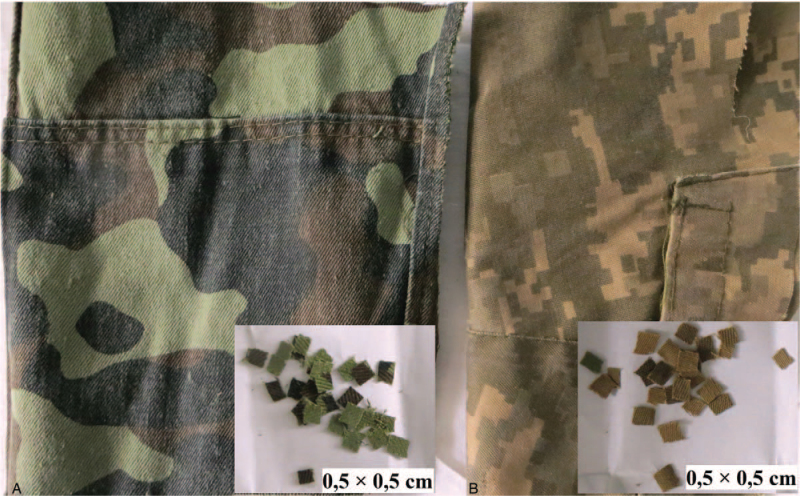
Implantable samples of military clothing: (A) the natural twill fabric, consisting of 100% cotton for experimental group 1 and (b) the fabric, consisting of 65% cotton, 35% polyester for experimental group 2.

As a sample of natural twill fabric, consisting of 100% cotton for experimental group 1, camouflage form “Dubok” was used (Fig. 1A). For experimental group 2, the 2015 release form, material 3403, consisting of 65% cotton, 35% polyester was used. The size of the implanted fragments was 0.5 × 0.5 cm (Fig. 1B).

### Surgical procedure

Animals’ anesthesia was performed with Zoletil (tiletamine hydrochloride and zolazepam hydrochloride) intramuscularly, which was administered at a dose of 10 mg/kg of animal body weight. The experiment was started after the onset of the surgical stage of anesthesia.

**Figure 2:**
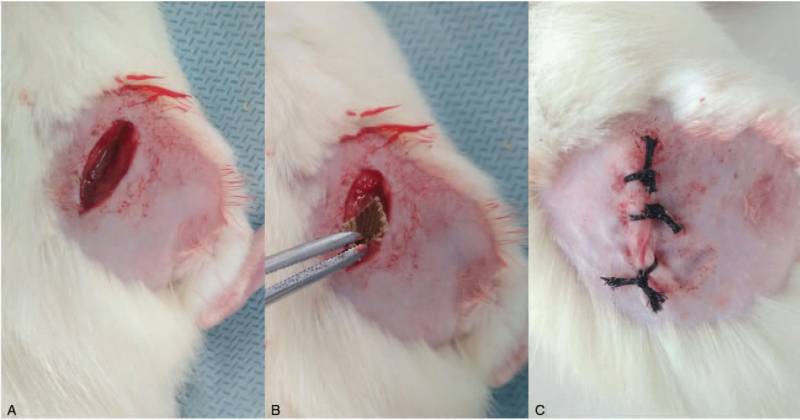
Surgical stages of implantation: (A) the incision of soft tissues of the posterior surface of the right thigh 1.0 cm long; (B) implantation in the muscle mass of textile fragment; (C) the sutured wound.

By randomization, the rats were divided into 3 equal groups of 18 animals each. Control group animals performed a layer-bylayer incision of soft tissues of the posterior surface of the right thigh 1.0 cm long with a surgical scalpel No. 11 with a partial incision of the muscles of the right thigh and subsequent suturing of the wound without implantation of foreign bodies. Animals of experimental group 1 were made a similar incision with the implantation of fragments of a fabric consisting of 100% cotton. Animals of experimental group 2 were made a similar incision with the implantation of fibers of a fabric consisting of 65% cotton and 35% polyester. Textile implantation in rats 1 and 2 of the experimental groups was carried out in the muscle mass of the posterior surface of the right thigh without damaging the bone and vascular structures, followed by suturing the wound. Wound suturing was carried out in layers with an atraumatic needle with 4/0 polypropylene suture, single-row interrupted surgical sutures (Fig. 2). All surgical operations were performed in a sterile environment in compliance with the rules of asepsis and antisepsis.

Wounds in all experimental animals healed quickly visually (up to 15 days). There was no suture divergence or signs of inflammation in the damaged area. The rats showed no signs of pain, they were quite active, so no analgesia was used. All animals were alive before being removed from the experiment. Removal of laboratory animals was carried out with the design of the study in equal groups of 18 animals (6 from each group) on the 15th, 30th, and 60th day after modeling the injuries with implantation of textile foreign bodies. Euthanasia was performed by an overdose of anesthesia.

### Histological evaluation

Soft tissue samples of experimental animals were histologically examined according to the standard methods. The material was fixed with 10% neutral formalin and dehydrated in ethanol solutions of increasing concentration: 50°, 70°, and *96*° (twice). Then it was carried out in alcohol with chloroform, chloroform, and embedded in paraffin. The sections made 5 to 7 μm thick were stained with hematoxylin and eosin, as well as Van Gieson picrofuchsin.^17^ The preparations were analyzed in a blinded manner and photographed using a PrimoStar microscope (Zeiss) and a Microocular digital camera.

## Results

### Histological evaluation after 15 days

After 15 days, in rats of the control group the wounds were completely epithelized. The newly formed epidermis was multilayered, with well-differentiated cells that made up the basal, thorny, granular, and horny layers. Under it, the defect area was made with maturing granulation tissue with bundles of collagen fibers oriented parallel to the wound surface, with a small number of capillaries. Its cellular composition was represented by fibroblasts and fibrocytes, single neutrophils, and lymphocytes. In the wound area adjacent to the intact dermis, the formation of hair follicles and sebaceous glands was observed.

**Figure 3:**
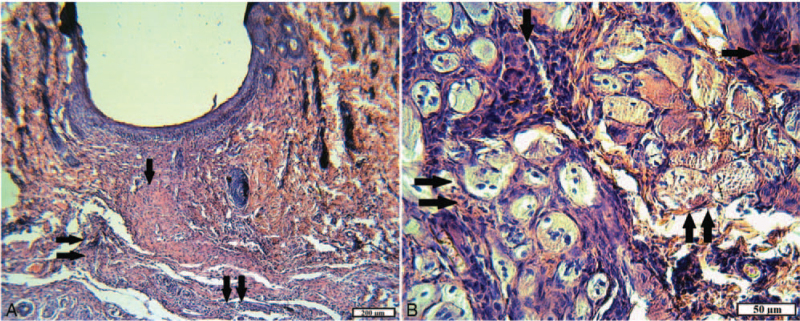
The plot of the wound channel of animals of the control group for 15 days. A, Complete epithelialization of the wound; ripening granulation tissue (arrow), edema, and foci of inflammation (double arrow) in the hypodermis (H&E). B, A portion of the muscle tissue adjacent to the wound channel: fibroblast proliferation (arrow); muscle fibers in a state of necrosis and necrobiosis (double arrow) (H&E).

In the deeper layers of the wound canal, in place of the damaged hypodermis and muscles, there was also maturing granulation tissue. However, leukocyte infiltration foci (with a predominance of neutrophilic granulocytes), macrophages, and isolated giant cells of foreign bodies were present here, which may indicate an active process of resorption of necrotic masses. Expanded vessels and cracks between the layers of young connective tissue were noted as manifestations of edema (Fig. 3A).

In the muscle tissue bordering the wound channel, signs of edema and venous capillary plethora were observed; the muscle fibers were dystrophic and necrotic. On destructively altered areas of muscle tissue, germination of connective tissue with a large number of fibroblasts with large, brightly colored functionally active nuclei was noted (Fig. 3B).

**Figure 4:**
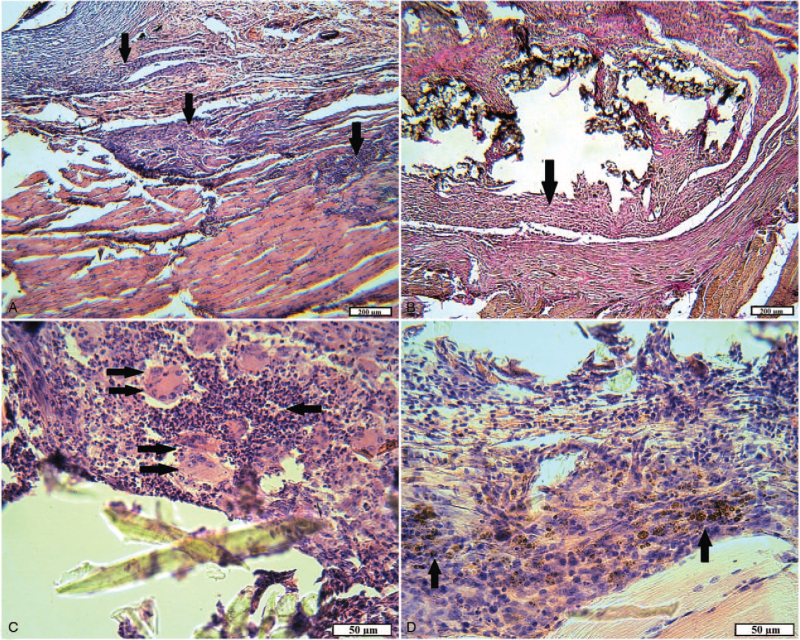
The plot of the wound of animals of the experimental group 1 for 15 days. A, A thin layer of the epithelium; ripening granulation tissue with pronounced leukocyte infiltration (arrow) and signs of edema (H&E). B, A double-layer capsule of a foreign body (arrow) (VGs). C, The area of the inner surface of the capsule: young granulation tissue with abundant neutrophil infiltration (arrow) and foreign body giant cells (double arrow) (H&E). D, Foreign body capsule: in the outer and inner layers, young granulation tissue with moderate leukocyte infiltration; numerous fibroblasts, hemosiderophages (arrow), capillaries (H&E).

In animals of experimental group 1 with implantation of tissue fibers consisting of 100% cotton, wounds epithelization was also complete after 15 days; however, the newly formed epidermis on the plots consisted of 1 to 3 layers of poorly differentiated cells, often with pyknotic nuclei and vacuolated cytoplasm, with cracks in the intercellular substance. Such a slowdown of regeneration may be associated with inflammatory processes occurring in the underlying layers of the wound channel. Severe diffuse and focal leukocyte infiltration, a large number of macrophages and giant cells of foreign bodies, fibroblasts, and dilated blood vessels were observed in the maturing granulation tissue, which replaces the wound defect of the dermis, subcutaneous fatty tissue, and muscles. In this case, the formed bundles of collagen fibers were loosely packed, had cracks and cracks, probably due to edema (Fig. 4A).

The capsules with implanted textile foreign bodies were located in the muscle tissue. Microscopically in the structure of the capsules stood out 2 layers. The outer layer of capsules consisted of granulation tissue, the maturation degree of which differed in different places. More “mature” sites with the presence of fibroblasts, with bundles of collagen fibers oriented parallel to the implant surface, with a small number of vessels were noted. A lot of capillaries, leukocytes, fibroblasts, and randomly arranged thin collagen fibers (Fig. 4B) were observed on the “younger” sites. The inner layer of capsules was uneven, with “outgrowths” into the cavity of the capsule and formed by young granulation tissue with pronounced inflammatory infiltration and a few “walled” textile fibers. In both layers, macrophages (including hemosiderophages) and giant cells of foreign bodies were detected. Thin-walled vessels of the capsule were full-blooded, often with perivascular hemorrhages. Textile fibers were observed in the lumen of the capsule, rounded in cross-section, or the form of a fuzzy polygon with a diameter of 30 to 35 mm, surrounded by fibrin, macrophage, leukocyte filaments, and tissue debris (Fig. 4C and D).

**Figure 5:**
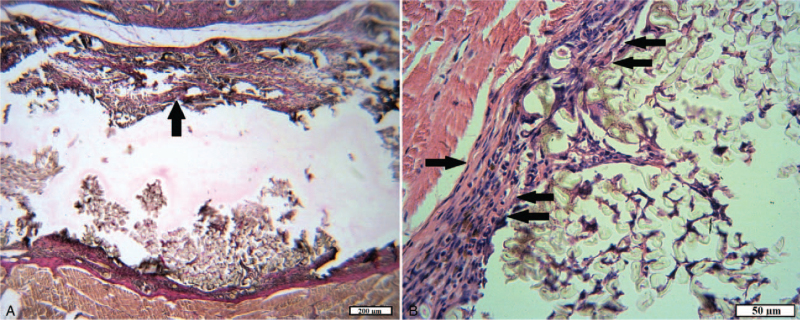
The site of the wound of animals of the experimental group 2 for 15 days. A, A double-layer capsule of a foreign body: the wall adjacent to the wound channel is thickened (arrow) (VGs). B, Foreign body capsule: tightly packed collagen fibers and fibrocytes in the outer layer (arrow); ripening granulation tissue with fibroblasts and few white blood cells - in the inner (double arrow); hemosiderin grains and giant cells of foreign bodies (H&E).

Microscopic examination of histopreparations of rats of experimental group 2 with the implantation of tissue fibers consisting of 65% cotton and 35% polyester showed results similar to those of experimental group 1 after 15 days. Differences were found in the structure of foreign body capsules. The capsules also consisted of 2 layers, but the thickness of their walls was significantly less in this group. The outermost layer of the capsule had the greatest thickness, directly adjacent to the wound canal, in which there were foci of the proliferation of fibroblasts and replacement of the damaged connective muscle tissue (Fig. 5A). Both layers were made with maturing granulation tissue with the orientation of bundles of collagen fibers parallel to the surface of the foreign body. Leukocyte infiltration of the capsule wall was weak in the outer layer and more pronounced, with macrophages and giant cells of foreign bodies in the inner layer. Expanded thin-walled vessels were noted, but no fresh hemorrhages were found in the thickness of the capsule. In some areas of the inner layer, hemosiderin grains were detected, which indicates hemorrhages that occurred earlier. In the cavity of the capsule, there were textile fibers of a crimped form 15 to 20 mm wide, surrounded by fibrin, leukocytes, and giant cells of foreign bodies. Part of the fibers was integrated into the wall of the capsule (Fig. 5B).

### Histological evaluation after 30 days

**Figure 6:**
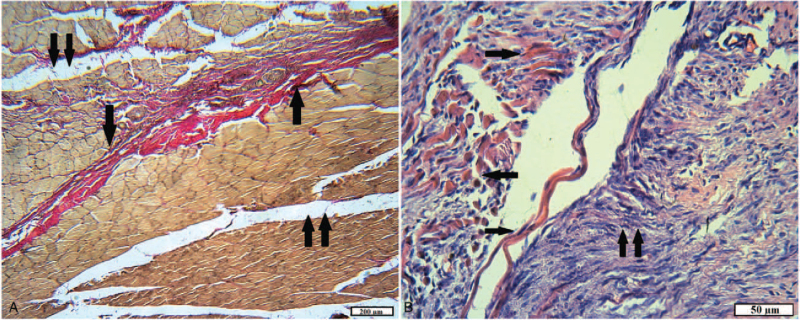
The plot of damaged muscle in rats of the control group after 1 month. A, Ripening connective tissue (arrow), signs of edema (double arrow) (VGs). B, Necrotic muscle fibers embedded in connective tissue (arrow); lysis of collagen fibers (double arrow), signs of edema, few white blood cells (H&E).

The study of histopreparations in the control group after 1 month showed complete recovery of the skin. Histoarchitecture of the epidermis and dermis corresponded to the norm. In the subcutaneous fatty tissue, layers of dense, mature connective tissue were determined. In the muscles, the process of maturation of the connective tissue continued, replacing destructively changed muscle fibers, fibroblasts numerically prevailed over fibroblasts, collagen fibers acquired dense packing. A decrease in the number of vessels and their differentiation was observed (Fig. 6A). At the same time, basophilic staining, delamination and lysis of collagen fibers were observed in the zone of the greatest damage to the muscles, where a wide scar was formed from mature granulation tissue, apparently due to the activation of fibroblasts at this stage of tissue repair. Also, signs of tissue edema and weak and moderate leukocyte infiltration were noted in some areas (Fig. 6B).

**Figure 7:**
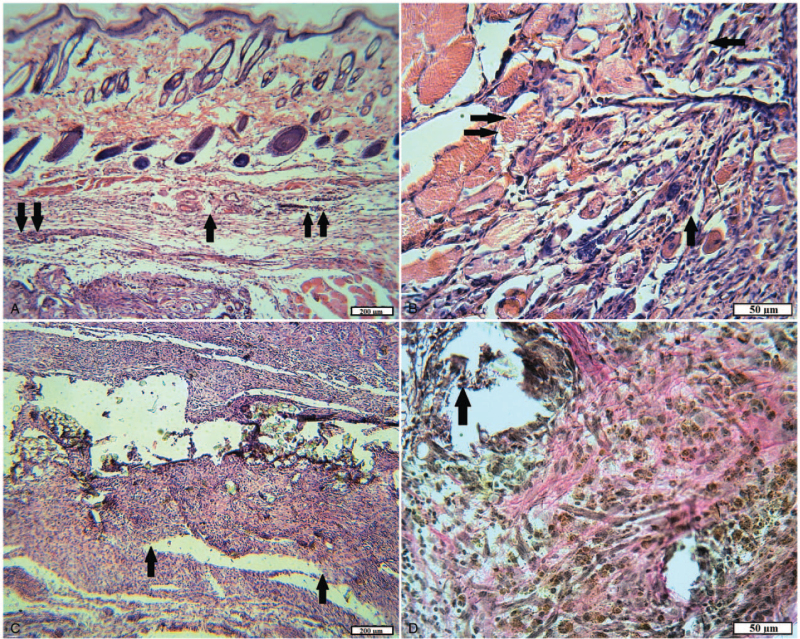
The site of the wound of animals of the experimental group 1 after 1 month. A, An animal skin area: the uneven thickness of the epidermis, signs of edema of all skin layers; dilated vessels (arrow), inflammation (double arrow) (H&E). B, Damaged muscle: fibroblast proliferation (arrow); muscle fibers in a state of necrosis and necrobiosis (double arrow); moderate inflammatory infiltration (H&E). C, Foreign body capsule: thickened walls with abundant inflammatory infiltration (arrow) (H&E). D, The inner part of the capsule: young granulation tissue is diffusely infiltrated with neutrophils and lymphocytes; capillaries; many siderophages; tissue detritus in the lumen of the capsule (arrow) (VGs).

In animals with implanted tissue fibers consisting of 100% cotton, after 1 month in histopathies, the epidermis was thinned or uneven in the areas with weak differentiation into layers. The hair follicles, the sebaceous glands, and the collagen stroma of the dermis were restored in the dermis, but all structures were “moved apart” due to edema. In the hypodermis, replaced by loose connective tissue, edema, dilated vessels, and focal and diffuse leukocyte infiltration were also observed (Fig. 7A). In the injured muscles, the process of replacement of necrotic and destructively changed muscle fibers with young connective tissue continued, as evidenced by the foci of fibroblast proliferation. In some areas, many myocytes in a state of necrosis and necrobiosis, in the form of “shadows,” were “walled up” in the connective tissue. Tissue swelling and inflammatory infiltration were also detected (Fig. 7B). The outer layer of the capsule consisted of ripening connective tissue with large vessels, the inner one–mainly of young granulation tissue with many capillaries. Both layers and surrounding tissues were densely infiltrated with inflammatory elements: neutrophils and lymphocytes, macrophages, and giant cells of foreign bodies. A small portion of the textile fibers was integrated into the capsule walls (Fig. 7C and D).

**Figure 8:**
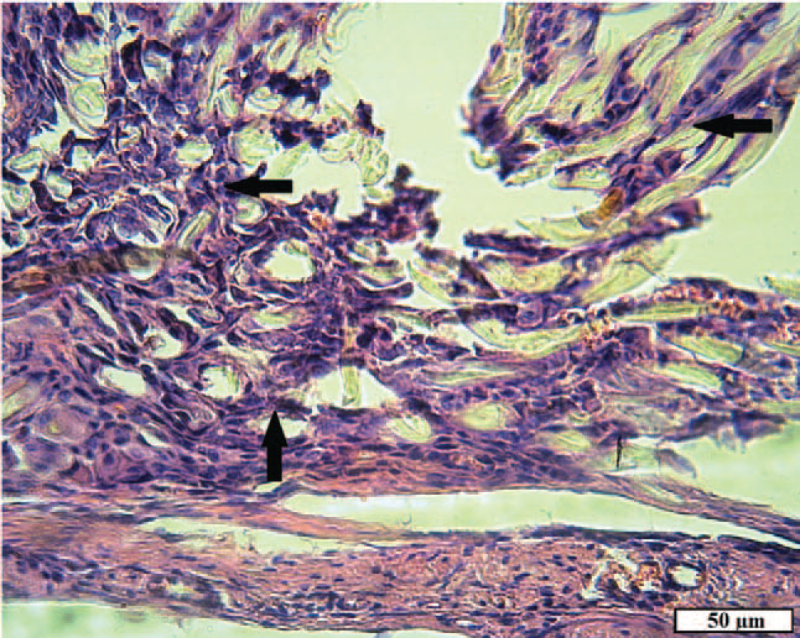
The inner layer and the cavity of the capsule of animals of the experimental group 2 after 1 month: young granulation tissue around the textile fibers (arrow) (H&E).

In animals of experimental group 2 with implantation of tissue fibers consisting of 65% cotton and 35% polyester, after 1 month, the repair of damaged tissues proceeded similarly to the previous group. Significant differences were moderate inflammatory reaction in all layers of the wound channel and the capsule, as well as the intensive formation of connective tissue around textile fibers (Fig. 8). The outer layer of the capsule was of different thickness in the areas and was formed by mature connective tissue with densely packed collagen fibers, with few differentiated vessels. In the inner layer, the arrangement of bundles of collagen fibers was predominantly chaotic; a moderate number of capillary type vessels and fibroblasts were noted.

### Histological evaluation after 60 days

**Figure 9:**
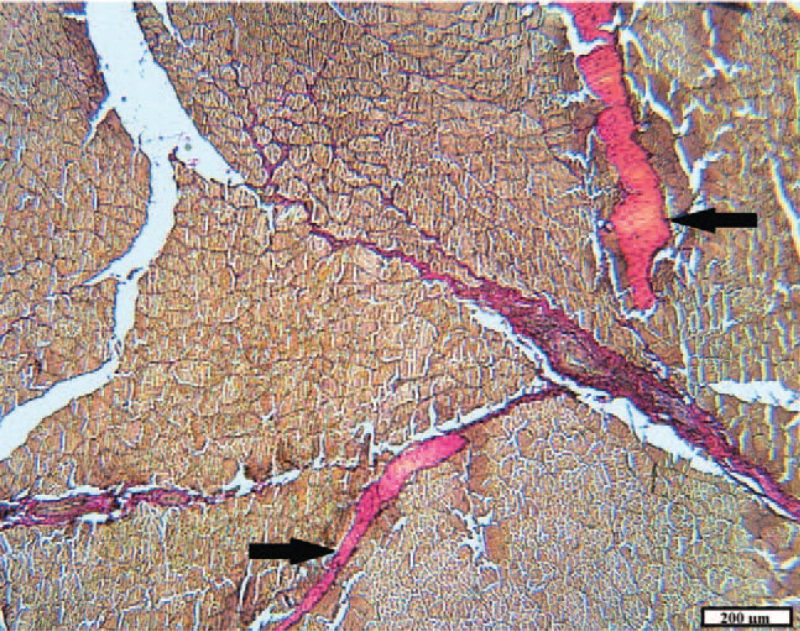
Cicatricial changes (arrow) in muscle tissue in animals of the control group after 2 months (VGs).

After 2 months in the control group, the reparative processes were completed. In the hypodermis and muscles, scars of dense, mature connective tissue with differentiated vessels were noted (Fig. 9). Myocyte regeneration was not observed, apparently due to extensive damage. Hyperplasia was noted (probably due to an increase in the number of myofibrils) of some muscle fibers near the scars, as a manifestation of a compensatory process.

**Figure 10:**
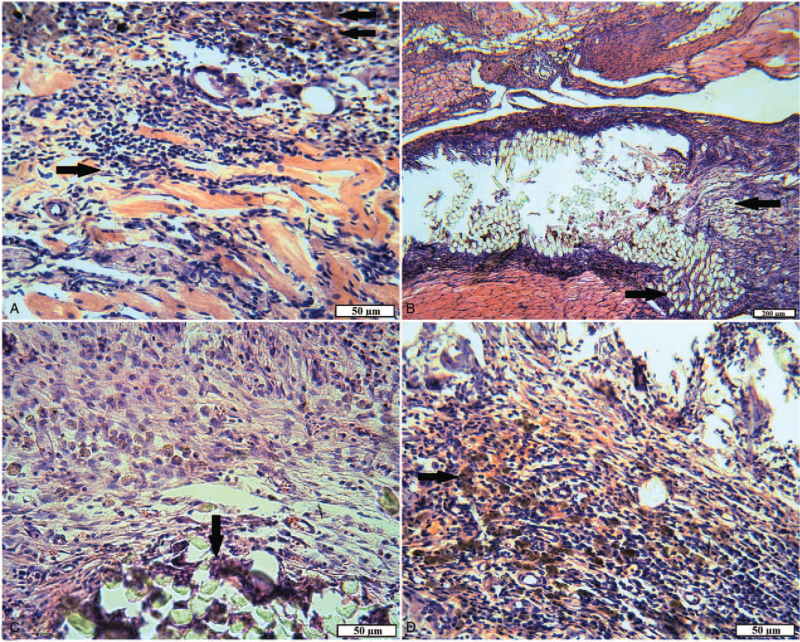
The site of the wound of animals of the experimental group 1 after 2 months. A, The area of damaged muscle: pronounced neutrophilic, lymphocytic infiltration (arrow) among destructively changed muscle fibers; accumulations of hemosiderophages (double arrow) (H&E). B, Foreign body capsule: abundant inflammatory infiltration; some of the threads are “walled up” in granulation tissue (arrow) (H&E). C, Foreign body capsule: the growth of young granulation tissue around textile fibers (arrow); a small number of leukocytes and hemosiderophages (H&E). D, Foreign body capsule: marked infiltration with neutrophils and lymphocytes; hemorrhages and accumulations of hemosiderophages (arrow); dead leukocytes and tissue detritus (H&E).

In the experimental group 1 in rats with implanted tissue fibers consisting of 100% cotton, after 2 months a pronounced inflammatory infiltration of the walls of the capsule and its surrounding tissues was observed. It is least manifested in the skin–in the form of the presence of a few neutrophilic granulocytes. In the wound channel and damaged muscle tissue, inflammatory infiltration in the areas ranged from weak diffuses to pronounced and focal. The wound canal was filled with ripening and young granulation tissue at different sites, slight hemorrhages, and accumulations of hemosiderophages were noted (Fig. 10A). The outer layer of the foreign body capsule predominantly consisted of mature granulation tissue with large vessels often with thickened walls, as a manifestation of chronic inflammation, the inner layer–of young granulation tissue, abundantly infiltrated with neutrophils and lymphocytes, containing giant cells of foreign bodies and hemosiderophages tissue. Often the inner surface of the capsule was a layer of neutrophils at various stages of necrosis and necrobiosis. In animals, the growth of granulation tissue around textile fibers was observed in separate areas (Fig. 10B-D).

**Figure 11:**
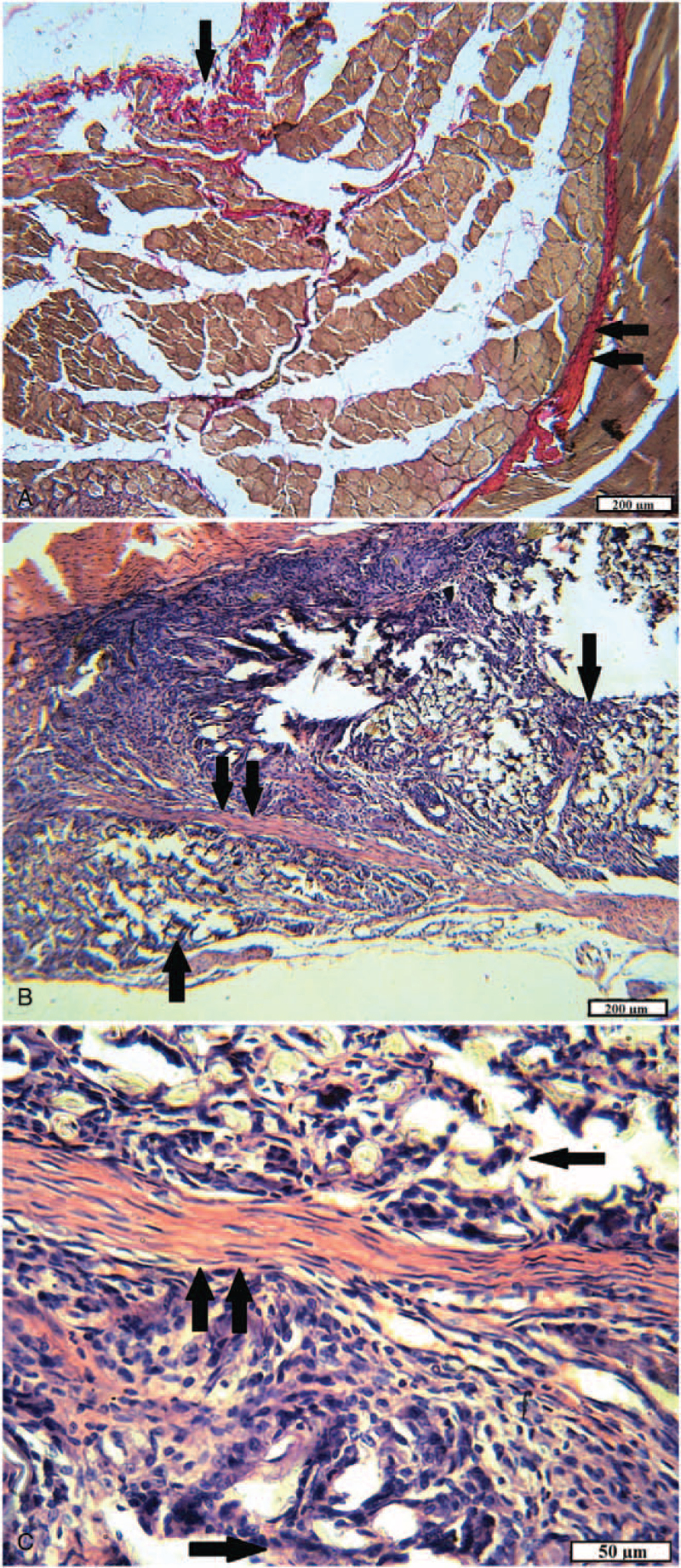
The site of the wound of animals of the experimental group 2 after 2 months. A, Damaged muscle: loose (arrow) and dense mature (double arrow) connective tissue (VGs). B, Foreign body capsule: dense connective tissue sheath; granulation tissue containing textile fibers (arrow); a layer of mature connective tissue inside the capsule (double arrow) (H&E). C, Foreign body capsule: granulation tissue with textile fibers (arrow); fibroblasts; a layer of mature connective tissue (double arrow) inside the capsule (H&E).

In the group of rats with implanted tissue fibers consisting of 65% cotton and 35% polyester, recovery of damaged tissues also continued after 2 months, but the inflammatory process was mild. In the damaged muscles, scars of dense and interlayers of loose mature connective tissue were found (Fig. 11A). Neutrophil granulocytes and lymphocytes in small quantities were found inside the capsule of a foreign body and in the tissues directly adjacent to it. The capsule wall consisted of mature connective tissue, bundles of collagen fibers were tightly packed, and fibrocytes were arranged between them, repeating the shape of the fibers. Inside the capsule was filled with young granulation tissue with a large number of fibroblasts, giant cells of foreign bodies, macrophages. It surrounded most of the implant fibers; a small number of fibers remained free in the central part of the capsule. In different directions, from one wall to another, the contents of the capsule were “lacerated” by layers of mature connective tissue of different thicknesses (Fig. 11B and C).

## Discussion

Wound healing in the majority of injuries follows an organized, partially coincident, 4-step process: activation of the blood coagulation cascade (hemostasis), inflammation, proliferation, and scar maturation or remodeling.^18^ Chronic wounds that do not follow the expected orderly healing path most often “get stuck” in the inflammatory phase, which leads to delayed wound healing.^19^

In all periods of the experiment in rats, the repair of damaged tissues was complicated by the presence of a textile implant. Differences between the groups were less noticeable for 15 days when the primary healing processes of granulation tissue of extensive damage of soft tissues, resorption of necrotic masses proceeded. In terms of 1 and 2 months, wound healing and scar remodeling in the experimental groups were slowed down due to the presence of inflammatory foci. A more pronounced inflammatory reaction was characterized by a group of animals with implanted tissue fibers consisting of 100% cotton. According to a semiquantitative analysis published in our previous work,^20^ the number of polymorphonuclear leukocytes in this group was 1.6 times higher at a period of 15 days, 3.1 times after 1 month, and 9.6 times after 2 months compared with the control group (*P* < .05). The dynamics of changes in the number of fibroblasts, vessels, and collagen reflected a slowdown in the growth and maturation of granulation tissue in the experimental groups, especially in group 1. This is probably due to the physicochemical properties of the fibers of this tissue (size, wall density, hygroscopicity, the presence of residues of protein molecules, etc) and made it difficult for phagocytosis and splitting of both partially the fibers and necrotic decay products, activated the release of leukocytes from the blood vessels, complicated the germination of connective tissue between the fibers. In the group with implanted tissue fibers consisting of 65% cotton and 35% polyester, the inflammatory reactions were less pronounced, probably due to the biological inertness of these textile fibers. The number of polymorphonuclear leukocytes in this group was 1.6 times higher at 15 days, 2.7 times after 1 month, 5 times after 2 months, compared with the control group (*P* < .05).^20^ By the end of the experiment, the implants were isolated from the surrounding tissues and “immobilized” by dense connective tissue membranes and layers in the capsule. This minimized the trauma of the muscles during movement and contact with the internal environment of the body, which reduced the possibility of reactivating the processes of inflammation.^21^,^22^

Macroscopically, all wounds in experimental animals healed well. In our opinion, this is due to the pronounced protective and reparatory mechanisms of the rat organism. At the same time, morphological studies revealed significant differences at the microscopic level in the healing of soft tissue wounds. The least pronounced local inflammatory reactions were observed in rats of the control group (without the presence of foreign bodies). More pronounced inflammation around foreign bodies was observed in rats 1 and 2 of the experimental groups. The greatest number of inflammatory reactions and their greatest severity was observed in animals of experimental group 1 in the presence of textile foreign bodies (fibers of the fabric consisting of 100% cotton), uniforms of military personnel. This can probably be explained by the absence of synthetic fibers in the tissue implanted in the wounds of animals of the experimental group 1. As is known from surgical practice, synthetic suture material (capron, prolene, polypropylene, polyglycolide) has greater biological inertness compared to natural suture threads (silk, polyester, catgut).^23^,^24^

## Conclusions

The presence of textile foreign bodies hampers the healing process of combat wounds of soft tissues due to the developing processes of inflammation around foreign bodies. The uniform of servicemen (35% synthetic and 65% natural fiber) is less reactive, leaving a wound as a textile foreign body, and has a less pronounced inflammatory effect, apparently due to the presence of synthetic threads that are more inert compared to fabric containing 100% natural fiber.

An important factor contributing to the healing of soft tissue wounds is an adequate surgical treatment with careful removal of textile foreign bodies, which creates conditions for a favorable course of the wound process.

## Acknowledgments

Assistance with the study: none.

## Conflicts of interests

The authors declare no conflicts of interest.
